# Polymorphisms in Autophagy-Related Gene* IRGM* Are Associated with Susceptibility to Autoimmune Thyroid Diseases

**DOI:** 10.1155/2018/7959707

**Published:** 2018-06-11

**Authors:** Qiu-ming Yao, Yuan-feng Zhu, Wen Wang, Zhen-yu Song, Xiao-qing Shao, Ling Li, Rong-hua Song, Xiao-fei An, Qiu Qin, Qian Li, Jin-an Zhang

**Affiliations:** ^1^Department of Endocrinology, Jinshan Hospital of Fudan University, No. 1508 Longhang Road, Jinshan District, Shanghai 201508, China; ^2^Department of Endocrinology and Nephrology, Weinan Central Hospital, Shaanxi Province 714000, China; ^3^Department of Urology, Jinshan Hospital of Fudan University, No. 1508 Longhang Road, Jinshan District, Shanghai 201508, China; ^4^Department of Endocrinology, The Affiliated Hospital of Nanjing University of Chinese Medicine, Nanjing 210023, China; ^5^Department of Endocrinology, Shanghai University of Medicine & Health Sciences Affiliated Zhoupu Hospital, No. 1500 Zhouyuan Road, Pudong District, Shanghai 201318, China

## Abstract

**Background:**

To date, studies have shown that polymorphisms in an autophagy-related gene,* IRGM*, are linked with different diseases, especially autoimmune diseases. The present study aimed to examine the roles of* IRGM* polymorphisms in autoimmune thyroid diseases (AITD).

**Methods:**

Three polymorphisms in* IRGM* gene (rs10065172, rs4958847, and rs13361189) were genotyped in 1569 participants (488 with Graves' disease, 292 with Hashimoto's thyroiditis, and 789 healthy controls) using PCR-based ligase detection reaction method. Gene-disease associations were evaluated for the three SNPs.

**Results:**

T allele of rs10065172, A allele of rs4958847, and C allele of rs13361189 were all higher in Graves' disease patients than controls, and the ORs were OR = 1.207 (*P* = 0.022), OR = 1.207 (*P* = 0.027), and OR = 1.200 (*P* = 0.027), respectively. After adjusting for sex and age, rs10065172 and rs13361189 were still associated with GD under both the allele model and dominant model, and the adjusted ORs for rs10065172 were 1.20 (*P* = 0.033) and 1.33 (*P* = 0.024), while the adjusted ORs for rs13361189 were 1.19 (*P* = 0.042) and 1.33 (*P* = 0.026), respectively. No significant difference was found between Hashimoto's thyroiditis patients and controls. Haplotype analysis found that CTA frequency was distinguishingly higher in Graves' disease patients (OR = 1.195, *P* = 0.030). The frequency of TCG haplotype was distinguishingly lower in AITD and Graves' disease patients (OR = 0.861, *P* = 0.044; OR = 0.816, *P* = 0.017).

**Conclusions:**

Our study reveals* IRGM* as a susceptibility gene of AITD and Graves' disease for the first time.

## 1. Introduction

Autoimmune thyroid disease (AITD) is the most prevalent autoimmune disease and mainly has two diverse types, Graves' disease (GD) and Hashimoto's thyroiditis (HT). AITD has a huge preponderance of females [1]. AITDs are characterized by lymphocytic infiltration responsive to thyroid antigens including thyroid-stimulating hormone receptor (TSHR), thyroid antigens including thyroglobulin (Tg), and thyroid peroxidase (TPO) [2]. In GD, anti-TSHR autoantibodies can bind to TSHR and stimulate the production and secretion of thyroid hormones, resulting in hyperthyroidism [3]. HT is characterized by the presence of thyroid autoantibodies against TPO and Tg [4].

Although the etiology of AITD remains elusive, the current paradigm is that the interplay between gene and environmental factors causes AITD [5, 6]. It is estimated that genetic factor accounts for about 70–80% of liability to the development of AITD [7]. The* HLA-DR* locus is the most important susceptibility gene involved in AITD [5]. Other identified susceptibility genes of AITD mainly have the following two groups: (1) immunity-related genes: protein tyrosine phosphatase-22 (*PTPN22*),* CD40*,* CTLA4, *and so on; (2) thyroid related genes: thyroglobulin (*TG*) and thyrotropin receptor (*TSHR*) genes [5].

Autophagy is an “autodigestive” process, which plays an important role in the delivery of intracellular components for terminal degradation and recycling [8]. This process has been associated with diverse aspects of innate and adaptive immunity [9], and abnormalities in the autophagic pathway have been involved in numerous autoimmune diseases, such as rheumatoid arthritis, systemic lupus erythematosus (SLE), and multiple sclerosis [10]. Immunity-related guanosine triphosphatase is critically important in defending against pathogens through regulating the progress of autophagy [11]. In the human genome, only immunity-related guanosine triphosphatase family M (*IRGM*) encodes a functional IRG among the three IRG genes (*IRGC, IRGQ*, and* IRGM*) [12]. IRGM is a key autophagy-related molecule, and it is also an important regulator involved in inflammation, which can prevent unwanted inflammation and protect cells from oxidative stress by regulating the engulfment of apoptotic cells [13]. Also, it can regulate the levels of cytokines, such as IL-1*β* and interferon gamma (INF-*γ*), through affecting the signaling pathway of toll-like receptor (TLR) [14]. To date, several polymorphisms in IRGM gene are reported to be linked with different diseases, especially autoimmune diseases such as inflammatory bowel disease (IBD) and SLE [10]. However, it is unknown whether* IRGM* gene variations are associated with AITD. Therefore, we conducted a case-control study to clarify this important issue.

## 2. Methods

### 2.1. Patients and Controls

In this study, a total of 780 patients with AITD (488 with GD and 292 with HT) were enrolled consecutively from the Outpatient Department of Endocrinology of Jinshan Hospital of Fudan University from November 1, 2012, to December 31, 2016, and 789 healthy controls were from the Health Check-Up Center of the same hospital during the same period. All subjects were south descendants of China. Individuals having other obvious chronic diseases were excluded from patient group. Individuals with thyroid disease or any other autoimmune disease were excluded from control group. Informed consent was obtained from all participants.

GD was diagnosed with both clinical findings of hyperthyroidism and laboratory data, such as decreased TSH value, elevated thyroid hormones or anti-thyroid-stimulating hormone receptor antibody (TRAb), anti-thyroid peroxide antibody (TPOAb), and anti-thyroglobulin antibody (TgAb). The diagnosis of HT was established by positive status of anti-thyroid peroxide antibody (TPOAb) or anti-thyroglobulin antibody (TgAb).

### 2.2. DNA Extraction

We collected 1 ml of peripheral venous blood in a tube containing ethylene diamine tetraacetic acid (EDTA) from each participant. The genomic DNA from peripheral blood was isolated using RelaxGene Blood DNA System (Tiangen Biotech Company, Beijing, China) according to the manufacture's protocol. The purity and concentration of all DNA samples were then detected.

### 2.3. SNP Selection and Genotyping

Three SNPs (rs10065172/rs4958847/rs13361189) of* IRGM* gene were selected from the Hapmap CHB data [15] with the following criteria: minor allele frequency (MAF) > 0.05, Hardy–Weinberg equilibrium (HWE) with *P* > 0.001, and logarithm of odds (LOD) > 3.0. These three SNPs were genotyped by PCR-based ligase detection reaction (LDR) method used in our previous studies [16–18]. The PCR condition was initial denaturation at 95°C for 2 min, denaturation at 94°C for 30 s, annealing at 62°C for 90 s, extension at 72°C for 1 min with 40 cycles and final extension at 65°C for 10 min. Primer sequences of the targeted loci are as follows:

  rs10065172
    Forward: CCCTTCGAAACACAGGACAT
  Reverse: TCATGCTGAATTGTGCAGAT
  rs4958847
    Forward: TGGATCCATCCATTTTCAACT
  Reverse: TTCCAAAATATTTGGTAGTCATGC
  rs13361189
    Forward: TGTCGTACCCAAGCAGAGTG
  Reverse: TCTAAACTGTACCCGCTGAG

The LDR was performed using denaturation at 95°C for 2 min, 40 cycles of annealing at 94°C for 15 s, and extension at 50°C for 25 s.

### 2.4. Statistical Analysis

Data were expressed as mean ± SD. We used Chi-square (*χ*2) test to evaluate the differences in the frequencies of alleles, genotypes and haplotypes among different groups, and the odds ratios (OR) were also calculated out. HWE test, linkage disequilibrium (LD) test, and haplotype frequencies were analyzed using Haploview 4.2. Logistic regression analysis was also performed, and age and gender were used as confounding factors in the multivariate logistic regression analysis. SPSS (Version 17.0) was used for statistical analysis and *P* value less than 0.05 suggested significant outcomes.

## 3. Results

### 3.1. Clinical Data

The clinical data of all subjects were shown in Table 1. In this study, we investigated 780 AITD patients (24.10% male and 75.90% female, mean age: 35.83 years), including 488 GD patients (30.53% male and 69.47% female, mean age: 36.65 years) and 292 HT patients (13.36% male and 86.64% female, mean age: 34.45 years). About 22.82% of AITD patients, 23.36% of GD patients, and 21.92% of HT patients had family history. The percentage of AITD, GD, and HT patients with TPOAb positive was 70.77%, 66.39%, and 78.08%, respectively, while the percentage of AITD, GD, and HT patients with TGAb positive was 80.51%, 79.10%, and 82.88%, respectively. The mean age of 789 healthy controls (34.35% male and 65.65% female) was 39.71 years.

### 3.2. Allele and Genotype Analyses

The genotype distributions of* IRGM* SNPs (rs10065172/rs4958847/rs13361189) were in HWE in both the case groups and control group (*P* > 0.05).  Table 2 showed the data of* IRGM* gene polymorphisms in AITD cases and controls. No distinguishing difference was found in both allele and genotype distributions of the three loci between cases and controls (*P* > 0.05).

The distributions of genetic frequencies of* IRGM* polymorphisms in GD patients, HT patients, and controls were shown in Table 3. In GD patients, the genotype distributions of the rs10065172 and rs13361189 showed a marginally significant trend compared with those of the controls, and *P* was 0.052 and 0.056, respectively. For rs4958847, no distinguishing difference in the genotype of* IRGM* polymorphisms was found between GD cases and controls. In GD patients, the minor T allele of rs10065172 was higher than controls (45.70% versus 41.06%, OR = 1.207, 95% CI = 1.027-1.418, *P* = 0.022,). For rs4958847, the minor allele A frequency was significantly higher in GD groups than controls (65.57% versus 61.22%, OR = 1.207, 95% CI = 1.022-1.425, *P* = 0.027). GD patients also had an increased frequency of allele C in rs13361189 compared with the controls (45.59% versus 41.13%, OR = 1.200, 95% CI = 1.021-1.409, *P* = 0.027). As Table 4 showed, after adjusting for sex and age, rs10065172 and rs13361189 were still associated with GD under both the allele model and dominant model, and the adjusted ORs for rs10065172 were 1.20 (*P* = 0.033) and 1.33 (*P* = 0.024), while the adjusted ORs for rs13361189 were 1.19 (*P* = 0.042) and 1.33 (*P* = 0.026), respectively. However, there was no distinguishing difference between HT patients and controls.

### 3.3. Haplotype Analysis

Three haplotypes were detected in the* IRGM* gene, including CTA, TCG, and TCA. As Table 5 displayed, in AITD patients, only the frequency of TCG haplotype was significantly lower than that in controls (35.22% versus 38.75%, OR = 0.861, 95% CI = 0.745-0.996, *P* = 0.044). Compared with controls, CTA haplotype had an increased frequency in GD patients (45.28% versus 40.98%, OR = 1.195, 95% CI = 1.017-1.404, *P* = 0.030). In contrast, the TCG haplotype was lower in GD cases (33.98% versus 38.75%, OR = 0.816, 95% CI = 0.690-0.964, *P* = 0.017). However, all the three haplotypes were not associated with HT.

### 3.4. Genotype and Clinical Phenotype Correlations

No correlation was found between the genotype or allele variants of the three tested SNPs and the clinical phenotypes, including family history, positive or negative antibodies (TGAb and TPOAb), thyroid size, and treatment outcomes (*P* > 0.05) (data not shown).

## 4. Discussion

IRGM plays an important role in autophagy [19].* IRGM* gene can induce autophagy by encoding a GTP-binding protein [14]. IRGM inhibition can result in impaired autophagy [11].* IRGM* genetic polymorphisms were confirmed to be related to many kinds of inflammatory and autoimmune diseases. Song et al. showed that* IRGM* rs10065172 was related to active tuberculosis in a Korean population [12]. Lu et al. reported that* IRGM* SNPs rs10065172 and rs13361189 were protective factors against latent TB progression in a Chinese population [20]. The polymorphism of* IRGM* rs13361189 was found to be related to leprosy by affecting the production of IL-4, IL-6, and INF-*γ* [21]. Our previous genetic studies also found that variants of inflammation and cytokine related genes were associated with AITD (Supplementary Table 1). Significant correlation between Crohn's disease (CD) and the C allele of rs13361189 (OR= 1.33) of the* IRGM* gene was noted in an Indian Population [22]. Besides, a recent study suggested IRGM polymorphisms were related to gastric cancer in Chinese [23]. In accordance with previous findings,* IRGM* rs13361189 and rs4958847 polymorphisms were confirmed to be crucial for CD susceptibility and phenotype modulation [24]. Interestingly, it was reported that the polymorphisms of* IRGM* gene were involved in susceptibility to SLE [25].

The present study is the first to investigate the relationship between the* IRGM* SNPs (rs10065172, rs4958847, and rs13361189) and susceptibility to AITD. Our allele and genotype results showed that T allele of rs10065172, A allele of rs4958847, and C allele of rs13361189 were higher in GD patients. After adjusting for sex and age, rs10065172 and rs13361189 were still associated with GD under both the allele model and dominant model, indicating that* IRGM* gene was associated with GD and might participate in the pathogenesis of GD and act as a risk factor. In GD, although the exact function of the* IRGM* SNPs is not fully elucidated,* IRGM* is possibly involved in the pathogenesis of GD because of its crucial role in autophagy, and autophagy has a vital function in innate and adaptive immunity. The polymorphism of* IRGM* gene may lead to the impaired autophagy, resulting in the disorder of immunity function, which needs to be explored by further studies. However, neither the allele nor genotype of rs10065172, rs4958847, and rs13361189 was related to HT, indicating that the three SNPs were not involved in the pathogenesis of HT. These different results of in the relationship* of IRGM* SNPs with GD and HT may be caused by their different etiology. Furthermore, haplotype analysis also suggested some roles of* IRGM* SNPs in AITD and GD.

In stratified analyses by clinical phenotype, no statistical significance of allele and genotype distributions was found between cases with each clinical phenotype and those without. The lack of significant findings may be because of the heterogeneity of environment or genetic factors in Chinese Han population and the relatively limited sample size caused by stratification. Further functional studies with large number of participants deserve continuing investigation.

In summary, our study is the first to suggest that* IRGM* rs10065172, rs4958847, and rs13361189 are related to GD in terms of both allele and haplotype frequencies, and TCG haplotype plays a protective role in AITD and GD. More studies with large number of participants are needed to further investigate the role of IRGM genetic polymorphisms in susceptibility to AITD.

## Figures and Tables

**Table 1:** Clinical data of all subjects.

	AITD	GD	HT	Control
N	780	488	292	789
Gender				
Male	188 (24.10%)	149 (30.53%)	39 (13.36%)	271 (34.35%)
Female	592 (75.90%)	339 (69.47%)	253 (86.64%)	518 (65.65%)
Age (mean ± s.d.)	35.83 ± 14.39	36.65 ± 14.73	34.45 ± 13.70	39.71 ± 8.34
Family history (+)	178 (22.82%)	114 (23.36%)	64 (21.92%)	-
Titer of antibodies				
TPOAb (+)	552 (70.77%)	324 (66.39%)	228 (78.08%)	-
TGAb (+)	628 (80.51%)	386 (79.10%)	242 (82.88%)	-
Thyroid size				-
Normal size	122 (15.64%)	83 (17.008%)	39 (13.36%)	-
I degree	135 (17.31%)	88 (18.033%)	47 (16.10%)	-
II degree	435 (55.77%)	247 (50.615%)	188 (64.38%)	-
III degree	88 (11.28%)	70 (14.344%)	18 (6.16%)	-

AITD, autoimmune thyroid disease; GD, Graves' disease; HT, Hashimoto's thyroiditis; TPOAb, anti-thyroid peroxide antibody; TgAb, anti-thyroglobulin antibody.

**Table 2:** Allele and genotype frequencies of IRGM SNPs in controls and AITD patients.

SNP	Genotype/Allele	Control (%)	AITD (%)	*P*	OR	95% CI
rs10065172	CC	275 (34.854)	238 (30.51)			
	CT	380 (48.162)	398 (51.03)	0.183	-	-
	TT	134 (16.984)	144 (18.46)			
	C	930 (58.94)	874 (56.03)	0.099	1.126	0.978-1.298
	T	648 (41.06)	686 (43.97)			
rs4958847	AA	293 (37.14)	325 (41.67)			
	AG	380 (48.16)	355 (45.51)	0.162	-	-
	GG	116 (14.70)	100 (12.82)			
	A	966 (61.22)	1005 (64.42)	0.063	0.872	0.754-1.008
	G	612 (38.78)	555 (35.58)			
rs13361189	TT	275 (34.85)	239 (30.64)			
	CT	379 (48.04)	398 (51.03)	0.205	-	-
	CC	135 (17.11)	143 (18.33)			
	T	929 (58.87)	876 (56.15)			
	C	649 (41.13)	684 (43.85)	0.124	1.118	0.970-1.288

AITD, autoimmune thyroid disease; OR, odds ratio; 95% CI, 95% confidence intervals.

**Table 3:** Allele and genotype frequencies of IRGM SNPs in GD, HT patients, and controls.

SNP	Control (%)	GD (%)	*P*	OR (95% CI)	HT (%)	*P*	OR (95% CI)
rs10065172							
CC	275 (34.854)	139 (28.48)			99 (33.90)		
CT	380 (48.162)	252 (51.64)	0.052	-	146 (50.00)	0.859	-
TT	134 (16.984)	97 (19.88)			47 (16.10)		
C	930 (58.94)	530 (54.30)	0.022	1.208 (1.028-1.419)	344 (58.90)	0.990	1.001 (0.826-1.214)
T	648 (41.06)	446 (45.70)			240 (41.10)		
rs4958847							
AA	293 (37.14)	210 (43.00)			115 (39.4)		
AG	380 (48.16)	220 (45.10)	0.081	-	135 (46.2)	0.792	-
GG	116 (14.70)	58 (11.90)			42 (14.4)		
A	966 (61.22)	640 (65.57)	0.027	0.829 (0.702-0.979)	365 (62.50)	0.586	0.947 (0.779-1.152)
G	612 (38.78)	336 (34.43)			219 (37.50)		
rs13361189							
TT	275 (34.85)	139 (28.50)			100 (34.25)		
CT	379 (48.04)	253 (51.80)	0.056	-	145 (49.66)	0.874	-
CC	135 (17.11)	96 (19.70)			47 (16.09)	0.874	-
T	929 (58.87)	531 (54.41)	0.027	1.200 (1.021-1.409)	345 (59.08)	0.932	0.992 (0.818-1.203)
C	649 (41.13)	445 (45.59)			239 (40.92)		

GD, Graves' disease; HT, Hashimoto's thyroiditis; OR, odds ratio; 95% CI, 95% confidence intervals.

**Table 4:** Association between SNPs of IRGM gene and AITD, GD, and HT by logistic regression analysis.

Disease	Model	Unadjusted *P*	Unadjusted OR (95% CI)	Adjusted *P*	Adjusted OR (95% CI)
AITD	rs10065172				
	Allele model	0.097	1.13 (0.98-1.30)	0.145	1.11 (0.96-0.98)
	Dominant model	0.067	1.22 (0.99-1.51)	0.108	1.19 (0.96-1.48)
	rs4958847				
	Allele model	0.063	0.87 (0.75-1.01)	0.204	0.91 (0.78-1.05)
	Dominant model	0.066	0.83 (0.68-1.01)	0.169	0.86 (0.70-1.06)
	rs13361189				
	Allele model	0.121	1.12 (0.97-1.29)	0.179	1.10 (0.96-1.28)
	Dominant model	0.076	1.21 (0.98-1.50)	0.122	1.19 (0.96-1.47)

GD	rs10065172				
	Allele model	0.021	1.21 (1.03-1.42)	0.033	1.20 (1.02-1.41)
	Dominant model	0.018	1.34 (1.05-1.72)	0.024	1.33 (1.04-1.70)
	rs4958847				
	Allele model	0.027	0.83 (0.70-0.98)	0.084	0.86 (0.73-1.02)
	Dominant model	0.036	0.78 (0.62-0.98)	0.104	0.82 (0.65-1.04)
	rs13361189				
	Allele model	0.026	1.20 (1.02-1.41)	0.042	1.19 (1.01-1.40)
	Dominant model	0.018	1.34 (1.05-1.72)	0.026	1.33 (1.03-1.70)

HT	rs10065172				
	Allele model	0.990	1.00 (0.83-1.22)	0.763	1.03 (0.84-1.27)
	Dominant model	0.771	1.04 (0.79-1.38)	0.709	1.06 (0.79-1.42)
	rs4958847				
	Allele model	0.585	0.95 (0.78-1.15)	0.783	0.97 (0.79-1.19)
	Dominant model	0.498	0.91 (0.69-1.20)	0.535	0.91 (0.68-1.22)
	rs13361189				
	Allele model	0.932	0.99 (0.82-1.20)	0.823	1.02 (0.83-1.26)
	Dominant model	0.852	1.03 (0.77-1.36)	0.774	1.04 (0.78-1.40)

AITD, autoimmune thyroid disease; GD, Graves' disease; HT, Hashimoto's thyroiditis; OR, odds ratio; 95% CI, 95% confidence intervals.

**Table 5:** Haplotype analysis of case groups and healthy control group.

Haplotype	Controls (%)	AITD	*P*	OR (95%CI)	GD (%)	*P*	OR (95%CI)	HT (%)	*P*	OR (95% CI)
CTA	645 (40.98)	679 (43.64)	0.132	1.115 (0.968-1.285)	441 (45.28)	**0.030**	1.195 (1.017-1.404)	238 (40.89)	0.975	0.997 (0.822-1.210)
TCG	610 (38.75)	548 (35.22)	**0.044**	0.861 (0.745-0.996)	331 (33.98)	**0.017**	0.816 (0.690-0.964)	218 (37.46)	0.568	0.944 (0.776-1.149)
TCA	318 (20.20)	321 (20.63)	0.734	1.031 (0.866-1.226)	197 (20.23)	0.989	1.001 (0.821-1.222)	124 (21.31)	0.560	1.072 (0.849-1.354)

AITD, autoimmune thyroid disease; GD, Graves' disease; HT, Hashimoto's thyroiditis; OR, odds ratio; 95% CI, 95% confidence intervals.

## Data Availability

The data analyzed during this study have been provided in the manuscript and any further information can be made available upon request to the corresponding author.

## Abbreviations

IRGM: Immunity-related guanosine triphosphatase family M
SNPs: Single nucleotide polymorphisms
AITD: Autoimmune thyroid disease
GD: Graves' disease
HT: Hashimoto's thyroiditis
TSHR: Thyroid-stimulating hormone receptor
Tg: Thyroglobulin
TPO: Thyroid peroxidase
CTLA4: Cytotoxic T lymphocyte-associated protein 4
PTPN22: Protein tyrosine phosphatase-22
IRGs: Immunity-related guanosine triphosphatase
TLR: Toll-like receptor
IBD: Inflammatory bowel disease
SLE: Systemic lupus erythematosus
TRAb: Anti-thyroid-stimulating hormone receptor antibody
TPOAb: Anti-thyroid peroxide antibody
TgAb: Anti-thyroglobulin antibody
EDTA: Ethylene diamine tetraacetic acid
MAF: Minor allele frequency
HWE: Hardy–Weinberg equilibrium
LOD: Logarithm of odds
PCR: Polymerase chain reaction
LDR: Ligase detection reaction
OR: Odds ratio
CI: Confidence interval
LD: Linkage disequilibrium
TB: Tuberculosis
INF-*γ*: Interferon gamma
CD: Crohn's disease
GC: Gastric cancer.

## Additional Points

*Highlights. *(i) Polymorphisms of rs10065172 and rs13361189 were associated with GD. (ii) The CTA haplotype was associated with GD. (iii) The TCG haplotype was associated with AITD and GD.

## Supplementary Materials

Supplementary Materials Our supplementary material was one table showing previous genetic studies on inflammation and cytokine related genes in AITD performed by our team.
