# Supplementary material for: Polymorphisms in Autophagy-Related Gene IRGM Are Associated with Susceptibility to Autoimmune Thyroid Diseases
Source: Biomed Res Int. 2018 Jun 11;2018:7959707. doi: 10.1155/2018/7959707 (PMC6016217; doi:10.1155/2018/7959707)
Supplement: Supplementary Materials — Our supplementary material was one table showing previous genetic studies on inflammation and cytokine related genes in AITD performed by our team. [file 7959707.f1.pdf]

Supplementary table 1. Previous genetic studies on inflammation and cytokine related genes in AITD performed by our team

Gene	Loci	cases	Controls	Association		
				AITD (P)	GD (P)	HT (P)
IL-37	rs3811046	1002	939	- (0.455)	- (0.062)	- (0.217)
	rs3811047			- (0.416)	- (0.052)	- (0.210)
	rs2723176			+ (0.044)	+ (0.014)	- (0.655)
	rs2723186			- (0.835)	+ (0.014)	- (0.685)
IL-17A	rs2275913	508	224	- (0.930)	- (0.862)	- (0.941)
	rs8193037			- (0.968)	- (0.985)	- (0.840)
	rs3819025			+ (0.000)	+ (0.011)	- (0.774)
IL-17F	rs763780	508	224	+ (0.000)	+ (0.002)	+ (0.001)
IL-21	rs907715	633	242	- (0.678)	- (0.963)	- (0.497)
	rs4833837			- (0.413)	- (0.606)	- (0.340)
	rs2221903			- (0.001)	+ (0.018)	+ (0.009)
	rs2055979			- (0.658)	- (0.782)	- (0.458)
IL-21R	rs3093301	633	242	- (0.606)	- (0.752)	- (0.138)
	rs2285452			- (0.359)	- (0.797)	- (0.068)
IL -22	rs2046068	975	851	- (0.278)	- (0.729)	+ (0.027*)
	rs2227478			- (0.339)	- (0.790)	+ (0.031*)
	rs2227485			- (0.341)	- (0.598)	- (0.232)
	rs1179251			- (0.901)	- (0.965)	+ (0.007*)
	rs11611206			+ (0.596)	- (0.364)	+ (0.765)

(P, P value of comparison of allele distributions between cases and controls; *, P value of comparison of allele distributions between male cases and male controls; AITD, autoimmune thyroid diseas; GD, Graves' disease; HT, Hashimoto's thyroiditis)
